# Four novel genetic mutations are associated with patent foramen ovale in Tibetan population using whole exome sequencing

**DOI:** 10.3389/fgene.2025.1592306

**Published:** 2025-08-18

**Authors:** Hongwei Li, Yongjun He, Yong Wu, Lanxin Liu, Wei Du, Duika Wang, Zeng He, Liming Zhao

**Affiliations:** ^1^ Department of Cardiology, Hospital of Chengdu Office of People’s Government of Xizang Autonomous Region (Hospital.C.X.), Chengdu, China; ^2^ School of Medicine, Xizang Minzu University, Xianyang, Shaanxi, China; ^3^ Biobank, Hospital of Chengdu Office of People’s Government of Xizang Autonomous Region (Hospital.C.X.), Chengdu, China

**Keywords:** pathogenetic mutations, whole exome sequencing, patent foramen ovale, Tibetan, genetics

## Abstract

**Objective:**

Patent foramen ovale (PFO), a prevalent congenital cardiac defect, is linked to clinical conditions such as cryptogenic stroke and migraine. The genetic underpinnings of PFO remain poorly elucidated, particularly in Tibet. This study aimed to identify potential pathogenic mutations in Tibetan PFO patients via whole exome sequencing (WES) to clarify its genetic basis.

**Methods:**

Eighteen Tibetan PFO patients diagnosed by echocardiography were enrolled. Peripheral blood samples underwent WES using Illumina HiSeq platform, followed by bioinformatics analysis to filter rare variants. Pathogenicity was assessed using predictive tools (SIFT, PolyPhen V2, and MutationTaster) and cardiac development-related gene databases (OMIM, HPO, HGMD, and MGI).

**Results:**

In this study, we identified four novel pathogenetic mutations in Tibetan PFO patients, including *GABRP* rs201584759 (c.421C>T: p. R141C), *GJB4* rs200602523 (c.292C>T: p. R98C), *RTTN* rs199568901 (c.5410G>A: E1804K), and *USH2A* rs144768593 (c.5608C>T: p. R1870W). Further analysis indicated that *GABRP*, *GJB4*, and *RTTN* were significantly associated with the occurrence of congenital heart disease.

**Conclusion:**

This study first reveals genetic characteristics of Tibetan PFO patients, implicating *GABRP*, *GJB4*, *RTTN*, and *USH2A* mutations in disrupting cardiac developmental pathways, potentially contributing to the occurrence of PFO. Findings underscore genetic factors regarding PFO prevalence in populations living in high-altitude and provide insights for molecular research and precision medicine.

## Introduction

Patent foramen ovale (PFO), a congenital cardiac structural defect resulting from incomplete embryonic closure of the foramen ovale, is pathologically characterized by a persistent interatrial channel between the left and right atria (Cho et al., 2021). This condition affects approximately 25% of the general population, though the majority remain clinically asymptomatic (Sposato et al., 2024). Interestingly, recent studies have reported higher PFO prevalence in specific populations. For instance, Möller et al. (2024) observed a PFO prevalence of 47% among Tibetans and Han Chinese living at high altitude (2,275 m), suggesting potential environmental or genetic influences on PFO occurrence (Möller et al., 2024). Notably, PFO has been implicated in diverse clinical sequelae through paradoxical embolism and right-to-left shunt mechanisms. Epidemiological evidence indicates that 40% of cryptogenic stroke patients exhibit concurrent PFO, underscoring its significant association with paradoxical embolic stroke (Mac Grory et al., 2022; Baik et al., 2022). Furthermore, this anatomical defect demonstrates clinical correlations with migraine and hypoxemia syndromes, and emerging research suggests potential involvement in atrial fibrillation and heart failure pathogenesis (Nguyen et al., 2024; Yan and Li, 2021; Mohan and Litwin, 2023). Regarding genetic mechanisms, genome-wide association studies (GWAS) have identified critical genes involved in angiogenesis and cardiac septation, including *TBX5* and *GATA4*, where loss-of-function mutations may disrupt endocardial cushion remodeling and impair foramen ovale closure (Apostolos et al., 2024). However, substantial heterogeneity persists in current evidence for pathogenic PFO-associated variants, with an incomplete understanding of population-specific penetrance differences and phenotype-genotype correlations (Paolucci et al., 2021). Systematic elucidation of PFO’s genetic architecture holds translational significance, not only for constructing risk stratification models but also for identifying molecular targets for precision interventions (Yeh and DeFaria Yeh, 2021).

The Tibetan population is predominantly distributed across the Tibetan Plateau and adjacent high-altitude regions in China, characterized by unique environmental adaptations and distinct genetic profiles. Studies show a significant link between high altitude and higher rates of congenital heart disease (CHD), with incidence rising as elevation increases (González-Andrade, 2020). Due to their exposure to relatively isolated environmental stressors and specific physiological traits, cardiovascular health issues in Tibetans—particularly those associated with PFO—remain understudied. Investigating the genetic determinants of cardiovascular diseases in this population holds significant scientific value as it provides critical insights into the interplay between environmental and hereditary factors shaping disease susceptibility within this ethnically distinct group.

Whole exome sequencing (WES), a high-throughput genomic technology, enables comprehensive analysis of protein-coding exonic regions in the genome. Compared to whole genome sequencing (WGS), this method demonstrates superior cost-efficiency for detecting coding sequence variations due to its targeted design, making it particularly valuable for identifying disease-associated genetic alterations (Morton et al., 2022). Consequently, WES has emerged as a critical tool in genetic research. Recent advancements in WES applications have significantly enhanced CHD investigations. This technology has facilitated the identification of monogenic pathogenic variants underlying various CHD subtypes, including hypoplastic left heart syndrome (Gordon et al., 2022), heterotaxy (Bolkier et al., 2022), and patent ductus arteriosus (Gao et al., 2022). Additionally, Li et al. (2024) demonstrated potential cardiovascular risk associations in PFO-related variants (Li et al., 2024). Notably, current genetic findings are predominantly derived from Han Chinese populations. The scarcity of WES data from Tibetan populations in high-altitude regions with elevated CHD prevalence creates substantial knowledge gaps, hindering cross-ethnic investigations into disease mechanisms and PFO-related genetic architecture.

This study employs WES technology to investigate genetic characteristics of PFO in Tibetan populations, focusing on identifying disease-associated mutations. The research seeks to provide novel insights into the genetic mechanisms underlying PFO development while establishing critical references for optimizing cardiovascular health strategies among ethnic Tibetan communities. Through comprehensive genomic analysis, we aim to reveal population-specific molecular markers that could enhance diagnostic precision and inform personalized prevention approaches for this cardiac defect.

## Materials and methods

### Study participants

This study enrolled 18 Tibetan patients with echocardiographically confirmed PFO from the Second People’s Hospital of Tibet Autonomous Region. Inclusion criteria comprised: 1) Diagnosis of PFO through transesophageal echocardiography (TEE) or contrast-enhanced transcranial Doppler (c-TCD); 2) Exclusion of other CHDs including atrial septal defect (ASD), ventricular septal defect (VSD) and patent ductus arteriosus; 3) No family history of inherited cardiovascular diseases in three-generation of direct relatives. Exclusion criteria were: 1) Comorbid acquired cardiac conditions (such as rheumatic heart disease or infective endocarditis); 2) Pulmonary hypertension (mean pulmonary artery pressure ≥25 mmHg as measured by right heart catheterization); 3) Cardiac structural/functional alterations secondary to systemic disorders (such as connective tissue diseases, thyroid dysfunction, chronic anemia); 4) Receiving catheter-based interventions within the last 3 months. Basic clinical data of the patients were collected from the hospital’s electronic medical records, as shown in Table 1. The research protocol received ethical approval from the Institutional Review Board of the Hospital of Chengdu Office of People’s Government of Xizang Autonomous Region (Hospital.C.X.) (Approval No.: Med-Eth-Re [2022] 77). Written informed consent was obtained from the legal guardians of all participants, and this study adhered to the ethical principles outlined in the Declaration of Helsinki throughout its process.

**TABLE 1:** Demographic and clinical data of PFO cases.

Sample ID	Age	Sex	Ultrasonic electrocardiogram report
COHD2_1	1 year and 6 months	women	CHD: PFO (2.5 mm) with a left-to-right shunt at the atrial level; mild tricuspid regurgitation
COHD13_1	2 years and 2 months	men	CHD: PFO post-surgery; CDFI: No shunt detected in the atria, ventricles, and large vessels
COHD16_1	3 years old	men	CHD: PFO; CDFI: Occasional left-to-right shunt observed at the atrial level
COHD19_1	2 years and 2 months	women	CHD: PFO (2.6 mm); CDFI: Left-to-right shunt observed at the atrial level
COHD26_1	3 years old	women	CHD: PFO (3 mm); CDFI: Left-to-right shunt observed at the atrial level; Mitral valve regurgitation (mild); Tricuspid valve regurgitation (small amount)
COHD27_1	8 years old	men	CHD: PFO (2 mm); CDFI: Left-to-right shunt observed at the atrial level; Tricuspid valve regurgitation (small amount)
COHD30_1	4 years old	women	CHD: PFO (2 mm); CDFI: Left-to-right shunt observed at the atrial level; Tricuspid valve regurgitation (small amount)
COHD31_1	12 years old	men	CHD: PFO (2.5 mm); CDFI: Left-to-right shunt observed at the atrial level; Tricuspid valve regurgitation (small amount)
COHD33_1	1 year and 2 months	men	CHD: PFO (2 mm); CDFI: Left-to-right shunt observed at the atrial level
COHD37_1	12 years old	women	CHD: PFO (2.5 mm); CDFI: Left-to-right shunt observed at the atrial level; Patent ductus arteriosus (5.5 mm); CDFI: Left-to-right shunt observed at the level of the great vessels; Tricuspid valve regurgitation (small amount)
COHD41_1	17 years old	women	CHD: PFO (3.5 mm); CDFI: Left-to-right shunt observed at the atrial level
COHD44_1	2 years and 7 months	men	CHD: PFO (2 mm); CDFI: Left-to-right shunt observed at the atrial level
COHD47_1	7 months	women	CHD: PFO (2.5 mm); CDFI: Left-to-right shunt observed at the atrial level
COHD53_1	8 years old	women	CHD: PFO (2.5 mm); CDFI: Left-to-right shunt observed at the atrial level; Tricuspid valve regurgitation (small amount)
COHD61_1	2 years and 6 months	men	CHD: PFO (3 mm); CDFI: Left-to-right shunt observed at the atrial level; Tricuspid valve regurgitation (small amount)
COHD67_1	2 years old	men	CHD: PFO (2 mm); CDFI: Left-to-right shunt observed at the atrial level; Tricuspid valve regurgitation (small amount)
COHD69_1	8 years old	men	CHD: PFO (3 mm); CDFI: Left-to-right shunt observed at the atrial level; Aortic valve regurgitation (small amount)
COHD71_1	2 days	women	CHD: PFO (3 mm); CDFI: Left-to-right shunt observed at the atrial level

CHD, congenital heart disease; PFO, patent foramen ovale; CDFI: color doppler flow imaging.

## WES

Genomic DNA was extracted from venous blood samples of PFO patients using the Gentra Puregene Blood Kit (QIAGEN, USA). The DNA quality was further evaluated by agarose gel electrophoresis, with the requirement for a clear main band and no tailing. DNA concentration (≥50 ng/μL, total ≥1.5 μg) and purity (OD260/280 = 1.8–2.0) were measured using a Nanodrop 2000. The DNA library was prepared by randomly fragmenting the DNA into 100–500 bp fragments with Covaris. Following end-repair and A-tailing, Illumina sequencing adapters were ligated to both ends of the library DNA using T4 DNA ligase. The library was then purified and size-selected using the Agencourt SPRLselect kit. Hybridization was performed in the solution phase with SureSelectXT Human All Exon V6 probes. Exon-targeted sequences were captured using streptavidin-coated magnetic beads, followed by PCR amplification. After the library passed quality control, it was further quantified via Qubit, and the insert size was verified by Agilent 2,100. The effective concentration of the library was precisely determined through qPCR (>5 ng/μL). Finally, the library was sequenced on the Illumina Hiseq platform in 2 × 150 bp paired-end mode to generate FastQ data.

### Mapping to human genome sequence, variants identification, and annotation

The quality of raw sequencing data was assessed using FastQC (v0.11.9), followed by low-quality read filtering (Phred quality score <20) and adapter removal using Trimmomatic (v0.39). The processed reads were aligned to the human reference genome GRCh37/hg19 using BWA-MEM (v0.7.17), and PCR duplicate reads were marked using Picard (v2.27.5). To further improve alignment accuracy, base quality score recalibration (BQSR) was performed using GATK (v4.2.6.1), and single-sample gVCF files, including single nucleotide variants (SNVs) and insertions/deletions (InDels), were generated using GATK HaplotypeCaller, following the GATK best practices.

### Priority classification for SNV/InDel

For all SNV/InDel variants, first apply the following filters: frequency in 1000Genomes, ExAC03 Asian population, and gnomAD Asian population must be below 0.01, GeneskyExonDB_Freq frequency must be below 0.05, and the variants should be non-synonymous or located in exonic regions. Then, classify the filtered variants into priority categories: First1 requires the variant to be either already present in HGMD or meet the following conditions: conserved (predicted as harmful by SIFT, BayesDel addAF), frequency in 1000Genomes below 0.001, frequency in ESP6500 below 0.01, SNV calling quality not equal to L, homogeneity equal to 1, and mutation frequency in Genesky Database below 0.005. First2 requires the variant to meet the following conditions: frequency in 1000Genomes below 0.001, frequency in ESP6500 below 0.01, SNV calling quality not equal to L, homogeneity equal to 1, and mutation frequency in Genesky Database below 0.005. Second is assigned to variants that have SNV calling quality not equal to L, frequency in 1000Genomes below 0.01, and homogeneity less than 3. Variants that do not meet any of the above conditions are classified as Third.

### Identification of pathogenic genes and variants

This study selects candidate pathogenic loci and genes based on the following criteria: First, priority is given to the First1 loci. Next, high-quality variants with an SNP Calling quality of “H” are selected. For functional prediction, loci predicted as damaging (D) by SIFT, PolyPhen V2, and MutationTaster are considered, with the additional requirement that the VEST Score is ≥0.5 to ensure the potential impact of the mutation on gene function. Finally, based on annotation data from databases such as OMIM, HPO, HGMD, and MGI, as well as functional annotations from GO and KEGG, further screening is carried out to identify candidate genes associated with diseases. We can efficiently identify potential pathogenic variants and related genes through this comprehensive filtering approach.

## Results

### Study patients

The basic characteristics of the participants are shown in Table 1. This study comprised 18 children with PFO, with balanced sex distribution (9 males/9 females) and ages spanning 2 days to 17 years. Echocardiographic measurements revealed PFO measuring 2.0–3.5 mm, with 94.4% (17/18) demonstrating persistent atrial-level left-to-right flow. Valvular dysfunction affected 72.2% (13/18) of cases, predominantly characterized by trivial/mild tricuspid regurgitation (61.1%, 11/18), with isolated mitral and aortic valve involvement observed in one case each.

### Comprehensive analysis of WES data

WES was performed on 18 PFO samples using the Illumina HiSeq platform, generating a total of 731,487,944 sequencing reads. The sequencing read lengths ranged from 15 to 151 bp. All samples exhibited high-quality clean reads with optimal Q20 and Q30 scores. The average proportion of bases with Phred quality score of ≥30 (Q30) in the raw data exceeded 97%, confirming the superior sequencing quality suitable for downstream bioinformatics analyses. Detailed statistical outcomes of the exome sequencing data are provided in Supplementary Table S1.

Alignment quality assessment of preprocessed reads from 18 samples was performed using the Picard toolkit (https://broadinstitute.github.io/picard/), with comprehensive metrics presented in Supplementary Table S2. The cohort demonstrated an average of 95,851,924.82 reads participating in genomic alignment, of which 60,767,983 reads (99.5% efficiency) were successfully mapped to the reference genome. Exome coverage analysis revealed that 98.3% of targeted regions achieved ≥10× depth, and 84.8% attained ≥30× depth on average. Following established guidelines, single nucleotide variants detected at positions with ≥10× coverage were considered reliably called.

**FIGURE 1:**
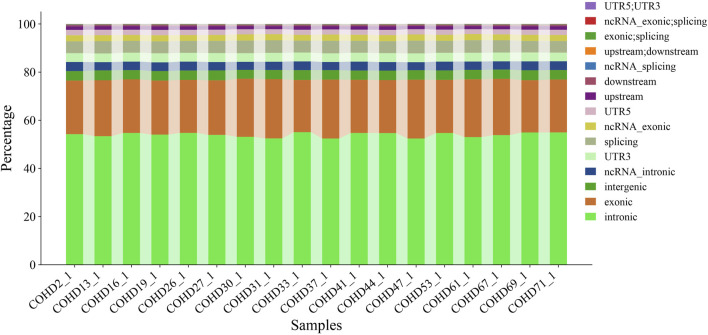
Variant distribution in genomic regions.

**FIGURE 2:**
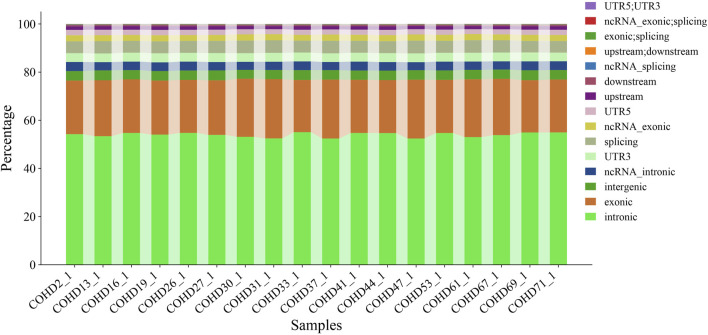
Distribution of functional types for all variants.

Using the GATK HaplotypeCaller pipeline, we identified 350,398 genetic variants comprising 291,532 single nucleotide variants (SNVs, 83.2%) and 58,866 insertion-deletion variants (InDels, 16.8%). Genomic region-based classification revealed distinct distribution patterns of SNVs/InDels across coding regions, regulatory elements, and intergenic regions, as illustrated in Figure 1. Functional annotation analysis further categorized these variants into missense, synonymous, and other functional types, with their respective proportions detailed in Figure 2. Systematic genotypic classification (homozygous/heterozygous) of both SNV and InDel loci was performed, with comprehensive quantitative data archived in Supplementary Table S3.

**FIGURE 3:**
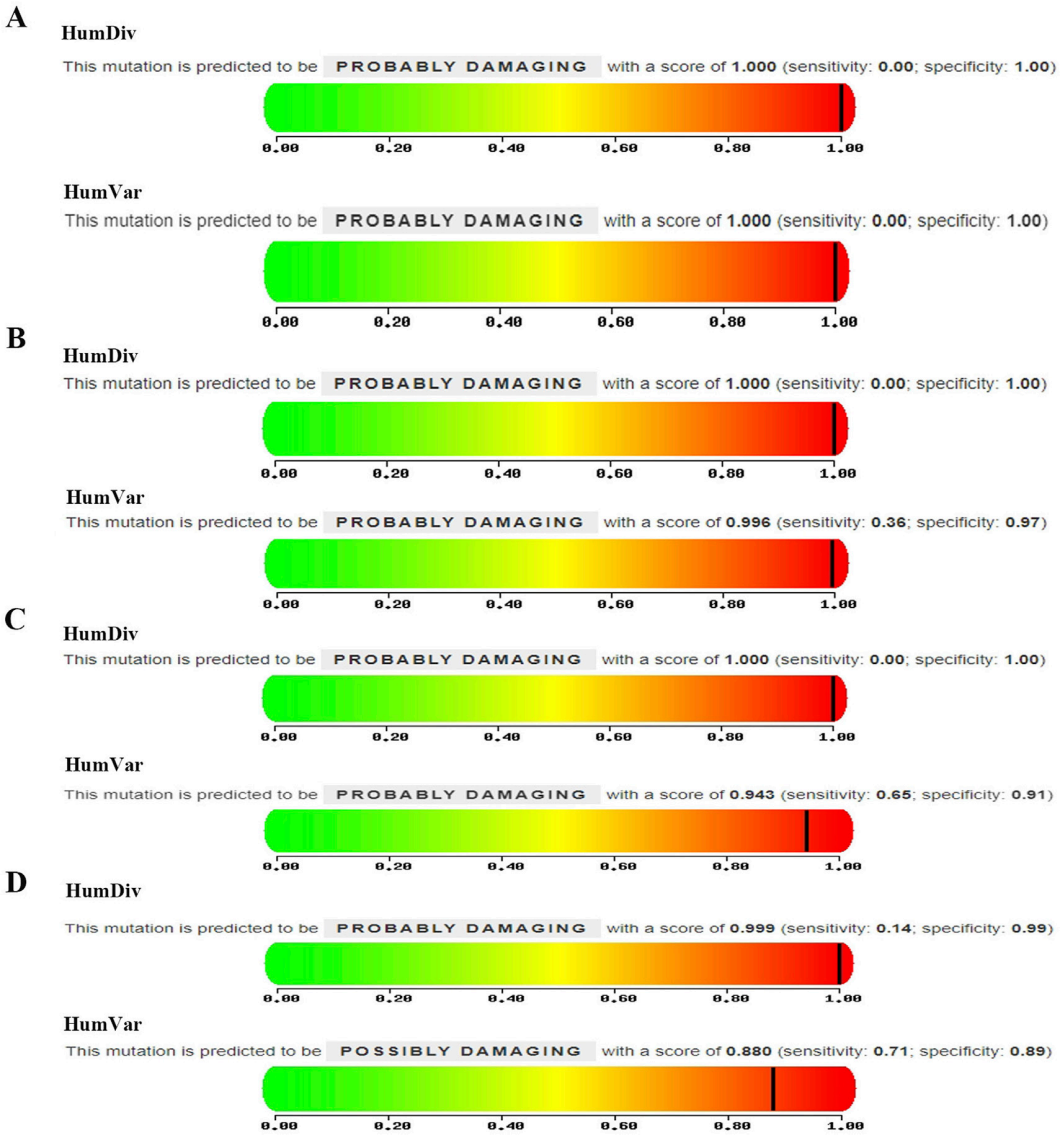
Mutation predictive of **(A)** *GABRP* (c.421C>T: p. R141C), **(B)** *GJB4* (c.292C>T: p. R98C), **(C)** *RTTN* (c.5410G>A: E1804K), and **(D)** *USH2A* (c.5608C>T: p. R1870W).

A multi-stage filtering pipeline was systematically applied to analyze detected SNVs/InDels. Initial prioritization identified 4,121 variants classified as “First 1″tier in the exome data of PFO patients. Subsequent filtering employed stringent population frequency thresholds: 1,000 Genomes <0.001, 1,000 Genome-EAS <0.01, ExAC03 < 0.01, gnomAD exome <0.01, and ESP6500 < 0.01, with concurrent requirement of high-quality SNP calling (grade H). This refined selection retained 28 rare variants. Pathogenicity prediction was performed using composite algorithms, including SIFT, PolyPhen-2, MutationTaster, and VEST3 (≥0.5 threshold) to identify deleterious mutations. Four exonic nonsynonymous SNVs were ultimately validated: *GABRP* (c.421C>T: p. R141C), *GJB4* (c.292C>T: p. R98C), *RTTN* (c.5410G>A: E1804K), and *USH2A* (c.5608C>T: p. R1870W). As illustrated in Figure 3, PolyPhen-2 predictions yielded pathogenic probabilities of 1.000 (*GABRP*), 0.996 (*GJB4*), 0.943 (*RTTN*), and 0.880 (*USH2A*), respectively. Besides, *GJB4* and *USH2A* were identified as pathogenic mutations through the HGMD database. The results of these four genes are presented in Table 2.

**TABLE 2:** Prediction of functional impairment impact on proteins resulting from four potential pathogenic genetic variants.

Gene	First priority	Freq_Alt (1000 g)	Freq_Alt (1000 g_eas)	ExAC03	esp6500	gnomAD_exome	SIFT Pred	POLYPhen V2 Pred	MutationTaster Pred	VEST score	Cadd Phred	Dann	HGMD_site_class
GABRP	First1	<0.001	0.002	<0.001	<0.001	<0.001	D	D	D	0.958	28.1	0.999	-
GJB4	First1	<0.001	0.003	<0.001	<0.001	<0.001	D	D	D	0.865	23.6	0.999	DM
RTTN	First1	<0.001	0.001	<0.001	<0.001	<0.001	D	D	D	0.557	24.6	0.999	-
USH2A	First1	<0.001	0.002	<0.001	<0.001	<0.001	D	D	D	0.856	25.0	0.999	DM

Freq_Alt (1000 g), alternative allele frequency data in 1000 Genomes Project; Freq_Alt (1000 g_eas), alternative allele frequency data in 1000 Genomes Project for EAS (East Asian); gnomAD, exome, Genome Aggregation Database exome; SIFT, pred, Sorting Intolerant From Tolerant Prediction; POLYPhen, V2 pred, Polymorphism Phenotyping v2 Prediction; Cadd Phred, Combined Annotation Dependent Depletion Prediction; Dann, Deleterious Annotation of Genetic Variants.

Table 3 shows detailed information of four mutation sites. These mutations are located in exonic regions and belong to non-synonymous SNVs, including rs201584759 in *GABRP*, rs200602523 in *GJB4*, rs199568901 in *RTTN*, and rs144768593 in *USH2A*. Additionally, functional annotation analysis indicated that *GABRP* was significantly associated with congenital heart disease and developmental disorder, while *GJB4* was involved in cardiomyopathy. *RTTN* was related to pulmonary stenosis, whereas *USH2A* was associated with amyotrophic lateral sclerosis (Table 4).

**TABLE 3:** Genetic data of predicted genes.

Gene	SNP ID	Chr	Position	Gene region	cDNA change	Protein change	InterVar	Functions
GABRP	rs201584759	chr5	170795388	exon5	c.421C>T	p.R141C	Uncertain significance	nonsynonymous SNV
GJB4	rs200602523	chr1	34761546	exon2	c.292C>T	p.R98C	Likely pathogenic	nonsynonymous SNV
RTTN	rs199568901	chr18	70048102	exon40	c.5410G>A	E1804K	Uncertain significance	nonsynonymous SNV
USH2A	rs144768593	chr1	216073265	exon28	c.5608C>T	p.R1870W	Uncertain significance	nonsynonymous SNV

SNP, single nucleotide polymorphism; Chr, Chromosome; cDNA, Complementary DNA; R, glutamine; E, ; K, ; H, histidine; C, cysteine; SNV, single nucleotide variant.

**TABLE 4:** Function analysis for the candidate genes.

Gene	OMIM	HPO	HGMD_gene	MGI	GO_BP	GO_MF	GO_CC	KEGG_Pathway
GABRP	-	-	congenital heart disease, developmental disorder	-	signal transduction, chemical synaptic transmission, ion transmembrane transport	GABA-A receptor activity, extracellular ligand-gated ion channel activity, chloride channel activity	integral component of plasma membrane, chloride channel complex, neuron projection	neuroactive ligand-receptor interaction - *Homo sapiens* (human)
GJB4	autosomal dominant, Erythrokeratodermia variabilis et progressiva 2	abnormality of cardiovascular system morphology, diabetes mellitus	cardiomyopathy	liver/biliary system phenotype, immune system phenotype, hematopoietic system phenotype	cell-cell signaling, transmembrane transport, gap junction-mediated intercellular transport	gap junction channel activity, protein binding	nucleoplasm, integral component of plasma membrane, connexin complex	-
RTTN	autosomal recessive	dysplastic corpus callosum, Agenesis of corpus callosum	Pulmonary stenosis	cardiovascular system phenotype	positive centriole replication, determination of left/right symmetry	-	cytoplasm, centrosome, ciliary basal body	-
USH2A	autosomal recessive, Usher syndrome, type 2A	autosomal recessive inheritance	amyotrophic lateral sclerosis	nervous system phenotype	tissue development, cell migration, substrate adhesion-dependent cell spreading	protein binding, collagen binding, myosin binding	photoreceptor inner segment, basement membrane, apical plasma membrane	-

## Discussion

PFO, a prevalent congenital heart defect, remains incompletely understood in its pathogenic mechanisms. Previous studies have shown that genetic factors likely play a significant role in PFO development (Moradi et al., 2023). Utilizing whole exome sequencing, we identified four novel pathogenic variants associated with PFO in the Tibetan population: rs201584759 (c.421C>T: p. R141C) in *GABRP*, rs200602523 (c.292C>T: p. R98C) in *GJB4*, rs199568901 (c.5410G>A: E1804K) in *RTTN*, and rs144768593 (c.5608C>T: p. R1870W) in *USH2A*. Functional investigations further elucidated the potential roles of *GABRP*, *GJB4*, and *RTTN* in CHD. These findings not only advance the understanding of genetic contributors to PFO in Tibetan populations but also illuminate molecular regulatory networks underlying cardiac developmental abnormalities in high-altitude hypoxic environments.

GABRP, a member of the neurotransmitter receptor family, has functions that extend beyond the traditional GABAergic signaling framework. Research reveals that GABRP orchestrates tumor microenvironment remodeling in pancreatic cancer by modulating KCNN4-dependent calcium ion flux, a mechanism independent of GABA transmission (Jiang et al., 2019). Similarly, GABRP sustains the self-renewal capacity of triple-negative breast cancer stem cells via EGFR pathway activation (Li et al., 2021), while also governing airway epithelial progenitor differentiation by regulating goblet cell formation, thereby maintaining tissue homeostasis (Wang et al., 2021). These findings underscore its pleiotropic regulatory roles in cellular signaling and developmental processes. Given its pivotal position in critical pathways, genetic alterations in GABRP may trigger diverse pathological consequences. For instance, GABRP polymorphisms correlate with susceptibility to systemic lupus erythematosus (Kim et al., 2015), and specific mutations potentially exacerbate immune checkpoint inhibitor-induced hepatotoxicity (Fontana et al., 2024). Notably, our study identified GABRP rs201584759 (c.421C>T: p. R141C) as a pathogenic variant in Tibetan patients with PFO, with functional analyses implicating its involvement in CHD. This evidence supports the hypothesis that GABRP mutations may critically contribute to PFO pathogenesis, though further experimental validation remains essential. Clinically, understanding the role of GABRP in calcium signaling pathways could provide insights into potential dysregulations in cardiac development relevant to PFO and point to pathways that might influence susceptibility to PFO-related complications like arrhythmias in the future.

The GJB4 gene encodes a connexin protein that regulates intercellular electrophysiological signaling by forming gap junctions, which are essential for maintaining cellular coordination and communication, particularly in tissues such as the skin and heart (Lucaciu et al., 2023; Dai et al., 2020). Research indicates that mutations in GJB4 are closely associated with various diseases, notably cutaneous and cardiovascular disorders. For instance, GJB4 mutations are linked to erythrokeratodermia variabilis et progressiva (EKVP), a skin condition characterized by impaired trafficking of connexins, leading to defective gap junction assembly (Zhang et al., 2022). Furthermore, GJB4 plays a critical role in cardiac function, where mutations or deficiencies may disrupt electrical signaling, contributing to arrhythmias and congenital heart defects (Okamoto et al., 2020). Our study has identified the GJB4 rs200602523 variant (c.292C>T: p. R98C) as a pathogenic mutation associated with PFO and cardiomyopathy, highlighting its systemic impact on cardiovascular health. The association of this specific variant with cardiomyopathy in our study underscores a direct clinical link between GJB4 mutations and significant cardiac phenotypes beyond PFO, suggesting that carriers of this variant, particularly in the Tibetan population, may warrant closer cardiac monitoring for potential functional impairment.

RTTN plays a critical role in cellular division, microtubule organization, and neurological development. It encodes a protein involved in essential biological processes such as spindle formation and stabilization, as well as cell cycle regulation (Kheradmand Kia et al., 2012). Mutations in RTTN disrupt these mechanisms, leading to diverse developmental disorders, particularly affecting the nervous and cardiovascular systems. For instance, RTTN variants are associated with primary microcephaly and primordial dwarfism, often manifesting as intellectual disability and developmental delays (Shamseldin et al., 2015). Additionally, RTTN is vital for cerebral cortex formation, with mutations causing structural abnormalities in cortical layering (Guguin et al., 2024). Beyond neurodevelopmental impacts, RTTN mutations may also impair cardiac and brain morphogenesis (Cavallin et al., 2018). Notably, studies link RTTN mutations to infantile dilated cardiomyopathy (IDC), where defective myocardial cell proliferation and differentiation result in cardiac dysfunction (Chun et al., 2023). Importantly, our investigation identified the RTTN rs199568901 variant (c.5410G>A: E1804K) as a pathogenic mutation in PFO. Those findings suggest that RTTN mutations may play a critical role in PFO pathogenesis by disrupting developmental pathways in cardiac septation. The established link between RTTN mutations and severe cardiomyopathies like IDC highlights the potential for RTTN variants, such as the one identified here, to confer risk for broader cardiac developmental issues or functional deficits, emphasizing the importance of comprehensive cardiac evaluation in individuals carrying such mutations.

USH2A encodes a protein critical for maintaining cellular structure and signaling, particularly in the auditory, visual, and cardiovascular systems. By stabilizing intercellular junctions, this protein supports retinal and inner ear development, with mutations linked to Usher syndrome (hearing and vision loss) and keratoconus (Ahmed et al., 2021; Yeo et al., 2025). Recent studies also associate USH2A mutations with congenital heart defects. For instance, rare variants (c.2299delG) were identified in fetuses with ventricular septal defects, suggesting disrupted cardiac cell adhesion or migration during development (Cao et al., 2022). Notably, our research identified pathogenic USH2A mutation (rs144768593, c.5608C>T: p. R1870W) patients with PFO, underscoring its broader role in cardiovascular anomalies. These findings emphasize the pleiotropic effects of USH2A mutations, though their specific mechanisms in cardiac disorders require further validation. The association of USH2A mutations with both structural heart defects (like VSD and now PFO) and sensory disorders (Usher syndrome, keratoconus) suggests that individuals diagnosed with PFO, especially if accompanied by sensory issues, could potentially benefit from genetic screening for USH2A variants, informing broader health management.

This investigation has several limitations. Primarily, the restricted sample size might diminish the broader applicability of the findings. Importantly, our cohort consisted exclusively of pediatric patients, and we recognize that the natural history of PFO closure dynamics differs significantly cross age groups. Besides, the present study is limited to 18 Tibetan pediatric PFO patients identified incidentally via cardiac murmur or routine physical examination; none exhibited clinical stroke or migraine. Consequently, subgroup or sensitivity analyses based on stroke or migraine phenotypes could not be performed. Validation of the association between the identified mutations and late-onset stroke or migraine will require larger, longitudinally followed adult Tibetan cohorts in future work. Additionally, while functional analyses provided initial insights into the association between identified genes and PFO, precise mechanistic pathways require validation through targeted experimental approaches. Notably, this research marks the inaugural discovery of four pathogenic mutations linked to PFO within the Tibetan population, offering novel perspectives on the genetic architecture underlying this cardiac defect. However, given the exploratory nature of this study and its focus on pediatric cases, it is critical to validate these findings in larger, independent cohorts spanning different age groups (especially adults) to confirm the mutations’ prevalence and their specific association with persistent PFO in the Tibetan population.

## Conclusion

This study offers critical genetic evidence for pathogenic mutations associated with PFO in Tibetan populations. The identification of four disease-causing variants (*GABRP* rs201584759, *GJB4* rs200602523, *RTTN* rs199568901, and *USH2A* rs144768593) may establish novel targets for early detection and personalized therapeutic strategies. Subsequent investigations should focus on elucidating the precise biological effects of these genetic alterations on cardiac morphogenesis and their underlying molecular pathways.

## Data Availability

The original contributions presented in the study are publicly available in the Figshare repository. This data can be found here: https://doi.org/10.6084/m9.figshare.29858897.v1.
