# Supplementary material for: Four novel genetic mutations are associated with patent foramen ovale in Tibetan population using whole exome sequencing
Source: Front Genet. 2025 Aug 18;16:1592306. doi: 10.3389/fgene.2025.1592306 (PMC12399560; doi:10.3389/fgene.2025.1592306)
Supplement: Supplementary file 1 [file Table1.docx]

Table S1 Exome sequencing data analysis

| Sample | R1 | | | | | R2 | | | | |
| --- | --- | --- | --- | --- | --- | --- | --- | --- | --- | --- |
|  | Total Reads | Reads length (bp) | Q20 | Q30 | BaseCount (M) | Total Reads | Reads length | Q20 | Q30 | BaseCount (M) |
| COHD2_1 | 59838401 | 15-151 | 99.97% | 97.77% | 8966.6 | 59838401 | 15-151 | 99.82% | 96.82% | 8951.12 |
| COHD13_1 | 44965060 | 15-151 | 99.96% | 97.35% | 6734.86 | 44965060 | 15-151 | 99.79% | 97.04% | 6723.11 |
| COHD16_1 | 58755327 | 15-151 | 99.97% | 97.68% | 8800.11 | 58755327 | 15-151 | 99.84% | 97.27% | 8785.76 |
| COHD19_1 | 59681159 | 15-151 | 99.97% | 97.68% | 8939.44 | 59681159 | 15-151 | 99.85% | 97.44% | 8924.13 |
| COHD26_1 | 63326823 | 15-151 | 99.97% | 97.67% | 9483.53 | 63326823 | 15-151 | 99.85% | 97.18% | 9468.04 |
| COHD27_1 | 37344706 | 15-151 | 99.97% | 97.52% | 5591.15 | 37344706 | 15-151 | 99.80% | 97.21% | 5582.18 |
| COHD30_1 | 22430116 | 15-151 | 99.98% | 98.18% | 3357.37 | 22430116 | 15-151 | 99.88% | 97.92% | 3352.21 |
| COHD31_1 | 21293930 | 15-151 | 99.98% | 98.34% | 3191.26 | 21293930 | 15-151 | 99.89% | 97.80% | 3186.23 |
| COHD33_1 | 55368506 | 15-151 | 99.98% | 97.79% | 8295.93 | 55368506 | 15-151 | 99.84% | 97.01% | 8282.67 |
| COHD37_1 | 24152275 | 15-151 | 99.98% | 98.33% | 3619.29 | 24152275 | 15-151 | 99.87% | 97.96% | 3613.61 |
| COHD41_1 | 50476381 | 15-151 | 99.97% | 97.81% | 7563 | 50476381 | 15-151 | 99.85% | 97.23% | 7550.78 |
| COHD44_1 | 54906826 | 15-151 | 99.97% | 97.77% | 8223.61 | 54906826 | 15-151 | 99.81% | 97.14% | 8210.72 |
| COHD47_1 | 26365164 | 15-151 | 99.98% | 98.24% | 3948.2 | 26365164 | 15-151 | 99.83% | 97.74% | 3942.33 |
| COHD53_1 | 48464924 | 15-151 | 99.98% | 97.95% | 7261.33 | 48464924 | 15-151 | 99.86% | 97.20% | 7249.72 |
| COHD61_1 | 21853464 | 15-151 | 99.98% | 98.21% | 3274.59 | 21853464 | 15-151 | 99.79% | 97.47% | 3269.45 |
| COHD67_1 | 26156534 | 15-151 | 99.98% | 98.38% | 3920.91 | 26156534 | 15-151 | 99.89% | 98.09% | 3914.74 |
| COHD69_1 | 56108348 | 15-151 | 99.98% | 97.88% | 8409.43 | 56108348 | 15-151 | 99.85% | 97.19% | 8395.7 |

Q20 and Q30 are sequencing data quality assessment metrics, representing the proportion of bases in raw data with quality scores ≥20 and ≥30, respectively.

Table S2 Calibration and coverage statistics in PFO patients

| Sample | BAIT_TERRITORY | Total reads | Reads mapped to the genome | Comparison ratio | Covered 10× | Covered 30× | Mean BAIT coverage |
| --- | --- | --- | --- | --- | --- | --- | --- |
| COHD2_1 | 60767983 | 111939362 | 60767983 | 0.995 | 0.992 | 0.905 | 167.13× |
| COHD13_1 | 60767983 | 134194188 | 60767983 | 0.995 | 0.995 | 0.910 | 201.69× |
| COHD16_1 | 60767983 | 118008138 | 60767983 | 0.996 | 0.991 | 0.910 | 169.55× |
| COHD19_1 | 60767983 | 130793980 | 60767983 | 0.995 | 0.987 | 0.885 | 203.71× |
| COHD26_1 | 60767983 | 106307082 | 60767983 | 0.994 | 0.994 | 0.894 | 159.74× |
| COHD27_1 | 60767983 | 55986736 | 60767983 | 0.994 | 0.939 | 0.698 | 82.35× |
| COHD30_1 | 60767983 | 136145714 | 60767983 | 0.995 | 0.995 | 0.898 | 208.08× |
| COHD31_1 | 60767983 | 44661442 | 60767983 | 0.996 | 0.970 | 0.709 | 64.61× |
| COHD33_1 | 60767983 | 44710574 | 60767983 | 0.996 | 0.973 | 0.737 | 66.57× |
| COHD37_1 | 60767983 | 136396156 | 60767983 | 0.995 | 0.994 | 0.937 | 207.54× |
| COHD41_1 | 60767983 | 48827988 | 60767983 | 0.996 | 0.973 | 0.722 | 71.62× |
| COHD44_1 | 60767983 | 98416762 | 60767983 | 0.995 | 0.993 | 0.895 | 150.76× |
| COHD47_1 | 60767983 | 95132410 | 60767983 | 0.994 | 0.994 | 0.901 | 147.04× |
| COHD53_1 | 60767983 | 45121364 | 60767983 | 0.996 | 0.936 | 0.646 | 67.67× |
| COHD61_1 | 60767983 | 119998208 | 60767983 | 0.995 | 0.993 | 0.925 | 174.38× |
| COHD67_1 | 60767983 | 104019454 | 60767983 | 0.995 | 0.994 | 0.926 | 151.14× |
| COHD69_1 | 60767983 | 98823164 | 60767983 | 0.995 | 0.992 | 0.921 | 141.49× |

Table S3 Statistics of SNVs/indels in all samples.

| Sample | SNV | InDel | Het_SNV | Hom_SNV | Novel_SNV | Het_InDel | Hom_InDel | Novel_InDel | Het_SNV/Hom_SNV |
| --- | --- | --- | --- | --- | --- | --- | --- | --- | --- |
| COHD2_1 | 96511 | 14237 | 56191 | 38421 | 1899 | 9142 | 3916 | 1179 | 1.46 |
| COHD13_1 | 93880 | 12888 | 53449 | 38473 | 1958 | 7987 | 3969 | 932 | 1.39 |
| COHD16_1 | 96365 | 15315 | 55612 | 38682 | 2071 | 9864 | 4089 | 1362 | 1.44 |
| COHD19_1 | 95446 | 14263 | 55076 | 38379 | 1991 | 9110 | 3916 | 1237 | 1.44 |
| COHD26_1 | 98806 | 15643 | 58238 | 38576 | 1992 | 10267 | 3972 | 1404 | 1.51 |
| COHD27_1 | 94463 | 13413 | 54059 | 38545 | 1859 | 8456 | 3908 | 1049 | 1.40 |
| COHD30_1 | 89085 | 11094 | 50524 | 36066 | 2495 | 6987 | 3351 | 756 | 1.40 |
| COHD31_1 | 88093 | 10461 | 50666 | 35745 | 1682 | 6427 | 3318 | 716 | 1.42 |
| COHD33_1 | 96859 | 15562 | 56382 | 38564 | 1913 | 10114 | 4176 | 1272 | 1.46 |
| COHD37_1 | 88104 | 10749 | 50495 | 35881 | 1728 | 6653 | 3349 | 747 | 1.41 |
| COHD41_1 | 97784 | 14998 | 57989 | 37784 | 2011 | 9829 | 3923 | 1246 | 1.53 |
| COHD44_1 | 96697 | 15450 | 54946 | 39734 | 2017 | 10005 | 4164 | 1281 | 1.38 |
| COHD47_1 | 86591 | 11209 | 50280 | 34664 | 1647 | 7230 | 3174 | 805 | 1.45 |
| COHD53_1 | 97496 | 15036 | 57706 | 37838 | 1952 | 9936 | 3857 | 1243 | 1.53 |
| COHD61_1 | 89817 | 11190 | 51710 | 36311 | 1796 | 7008 | 3439 | 743 | 1.42 |
| COHD67_1 | 91651 | 11986 | 52641 | 37264 | 1746 | 7545 | 3599 | 842 | 1.41 |
| COHD69_1 | 97604 | 15575 | 55541 | 39941 | 2122 | 10011 | 4204 | 1360 | 1.39 |

SNV: single nucleotide variation; InDel: insertion-deletion; HET: Heterozygous; HOM: Homozygous.
